# Is it financially beneficial for hospitals to prevent nosocomial infections?

**DOI:** 10.1186/s12913-020-05428-7

**Published:** 2020-07-14

**Authors:** Shmuel Benenson, Matan J. Cohen, Carmela Schwartz, Michael Revva, Allon E. Moses, Phillip D. Levin

**Affiliations:** 1 Department of Clinical Microbiology and Infectious Diseases, Hadassah-Hebrew University Medical Center, POB 12000, 9112001 Jerusalem, Israel; 2 Clalit Health Services, Jerusalem district, affiliated with the Hebrew University School of Medicine, Jerusalem, Israel; 3 Finance Department, Hadassah-Hebrew University Medical Center, Jerusalem, Israel; 4 Critical Care Unit, Shaare Zedek Medical Center, Jerusalem, Israel

**Keywords:** Nosocomial infection, Attributable cost, Central line associated blood stream infections, *Clostridium difficile* infection, Surgical site infection

## Abstract

**Background:**

Financial incentives represent a potential mechanism to encourage infection prevention by hospitals. In order to characterize the place of financial incentives, we investigated resource utilization and cost associated with hospital-acquired infections (HAI) and assessed the relative financial burden for hospital and insurer according to reimbursement policies.

**Methods:**

We conducted a prospective matched case-control study over 18 months in a tertiary university medical center. Patients with central-line associated blood-stream infections (CLABSI), *Clostridium difficile* infection (CDI) or surgical site infections (SSI) were each matched to three control patients. Resource utilization, costs and reimbursement (per diem for CLABSI and CDI, diagnosis related group (DRG) reimbursement for SSI) were compared between patients and controls, from both the hospital and insurer perspective.

**Results:**

HAIs were associated with increased resource consumption (more blood tests, imaging, antibiotic days, hospital days etc.). Direct costs were higher for cases vs. controls (CLABSI: $6400 vs. $2376 (*p* < 0.001), CDI: $1357 vs $733 (*p* = 0.047) and SSI: $6761 vs. $5860 (*p <* 0.001)). However as admissions were longer following CLABSI and CDI, costs per-day were non-significantly different (USD/day, cases vs. controls: CLABSI, 601 vs. 719, (*p* = 0.63); CDI, 101 vs. 93 (*p* = 0.5)). For CLABSI and CDI, reimbursement was per-diem and thus the financial burden ($14,608 and $5430 respectively) rested on the insurer, not the hospital. For SSI, as reimbursement was per procedure, costs rested primarily on the hospital rather than the insurer.

**Conclusion:**

Nosocomial infections are associated with both increased resource utilization and increased length of stay. Reimbursement strategy (per diem vs DRG) is the principal parameter affecting financial incentives to prevent hospital acquired infections and depends on the payer perspective. In the Israeli health care system, financial incentives are unlikely to represent a significant consideration in the prevention of CLABSI and CDI.

## Background

During the course of their hospital stay approximately 5–10% of patients develop a hospital acquired infection (HAI) [1]. Such infections are associated with morbidity, mortality, increased lengths of stay and costs [2–9].

Costs associated with HAIs are estimated to be up to $25,000 per infection [2]. However, nosocomial infections usually affect more severely ill patients, who often have long, complex and expensive hospital courses regardless. Estimates of attributable costs of nosocomial infections are often based on cohort or database analyses that do not directly compare the costs of HAI to the costs of admission for similarly complex patients. Furthermore, the origins of costs related to HAIs have not been clearly delineated.

The distribution of HAI related costs between the different institutions within the health care system – primarily the hospital and insurer – and the interaction with the source of costs and the reimbursement strategy have not been widely investigated. For example, per diem billing will affect the hospital and insurer in a different way relative to fixed payments associated with diagnosis related groups (DRG), and this may be further influenced in systems where the insurer owns the hospital. Analysis of distribution of costs is important as it might indicate how financial measures could be used as incentives to improve prevention of HAIs [10].

In this study we analyzed attributable resource utilization and costs associated with treatment of central line associated blood stream infections (CLABSI), *Clostridium difficile* infection (CDI) and surgical site infection (SSI) when compared to matched control patients without nosocomial infections and estimate how these expenses differ between the hospital and the insurer.

## Methods

Over 18 months during 2014 and 2015, consecutive patients with CLABSI, CDI or SSI were enrolled, each of whom was compared to three matched controls without infection. We measured treatments and equipment used, costs and clinical outcome. The study was performed at the Hadassah Medical Center in Jerusalem, Israel, a tertiary care academic medical center which comprises two campuses: one with 800 and the other with 350 beds.

Primary study outcomes were differences in resource utilization and costs between cases and matched controls. We also compared costs incurred by the hospital compared to medical insurers. Secondary outcomes included length of hospital stay, proportion of patients discharged to long term care facilities, readmissions and all-cause mortality.

The health care system described involves an independent hospital reimbursed by separate health care maintenance organizations on either a per diem of DRG basis.

### Study population

Active surveillance for the three specified infections was performed routinely and independently from the study protocol by infection control physicians and nurses. Presence of infection was defined according to Centers for Disease Control (CDC) criteria [11]. The study population included consecutive cases of CLABSI in intensive care units (ICU), CDI in adult medical and surgical wards, and SSI following orthopedic surgery (either hip, knee or back) and general surgery (either colon, gall bladder, breast or inguinal hernia repair). Patients with hematological malignancy or after bone marrow transplantation were excluded.

Each enrolled patient was matched to three patients without infection during their entire hospital stay (or for 3 months post-surgery for SSI) amongst patients in the same department around the same time. Matching was performed according to age groups, sex, ward, type of surgery for SSI and severity of illness (SAPS II [12] on admission to ICU for CLABSI, Charlson comorbidity index [13] at hospital admission for CDI and preoperative ASA score [14] for SSI). In addition, control patients for CLABSI and CDI cases were matched for length of admission prior to the appearance of infection. For CLABSI the inclusion date for matching the pre-infection length of stay was determined as 2 days prior to the positive blood culture defining CLABSI. For CDI the inclusion date for matching was 2 days prior to the appearance of diarrhea which was subsequently diagnosed as being caused by *Clostridium difficile.* SSI patients were not matched for length of stay prior to infection but rather from the day of surgery.

When more than three appropriate controls were identified, three were chosen randomly. If less than three controls were identified available control patients were used, while if no control patients could be identified, the case (infection) patient was excluded from further analysis.

Study follow-up continued until death or 90 days after hospital discharge (for survival and readmission).

### Data collection

Demographics, mortality, readmission, discharge destination and length of stay were recorded for all patients. Contributors to resource utilization included: blood, microbiological and pathological tests, diagnostic imaging, antibiotic and blood product administration, nutrition used, and all invasive procedures. These data were obtained from chart review and priced according to the purchase price paid or otherwise determined by the hospital administration. The same pricing process was used to evaluate costs for case and control patients. Fixed costs (such as salaries, electricity, maintenance etc.) were not included in the analysis.

#### Opportunity costs

Opportunity costs resulted from:

1. Increased reimbursement for ICU patients during the first 4 days of their ICU admission. Prolonged admission potentially decreased the availability of a bed for a new patient who would provide higher reimbursement for the first 4 days of his admission.
2. Diagnosis Related Groups (DRG): included surgical procedures were reimbursed on a DRG basis. Prolonged admissions related to HAIs thus potentially blocked beds leading to losses resulting from the inability to admit new DRG patients.

#### Readmissions

Costs associated with readmissions differed for CLABSI/CDI and SSI patients as a result of different reimbursement arrangements. For CLABSI/CDI readmissions, costs were reimbursed on a per diem basis identical to the first admission. For SSI patients (all of whom were reimbursed by DRG), readmission within 7 days after initial discharge was considered as part of the first admission without additional reimbursement, whilst readmission after 7 days from initial discharge was reimbursed on a per diem basis.

### Data analysis

Costs were collected and analyzed in the local currency (New Israeli Shekels, NIS). Data are however presented in United States Dollars (USD) using the exchange rate set by the bank of Israel on January 1st 2016, 1 USD = 3.913 NIS.

### Sample size

We estimated that, on average, treatment of a patient with HAI would cost the hospital $1400 more than a control case. Based on an estimated cost of $102 per day for 10 days of antibiotic treatment, and additional possible expenses, we assumed a standard deviation of $3800. With a power of 80% and a significance of 0.05 (alpha), the sample size required was 78 sets of one case and three controls. Of the three HAIs in our focus, the least frequent during 2012 was CLABSI (51 cases/year), and we thus calculated that during 18 months we would collect approximately 75 CLABSI cases.

Use of elements contributing to resource utilization and secondary outcomes were compared for cases and controls (three matched controls per a case) using the paired t-test (continuous variables) and the McNemar test (categorical variables). A *p*-value of 0.05 was considered significant.

Average differences in costs between cases and controls are presented as the main analysis. Total costs and costs per day were calculated and compared for cases and controls. A comparison of resource utilization was performed between the case and: (i) the mean of all three specific controls; (ii) the control with the highest associated costs/resource use; (iii) the control with the lowest costs/resource use, enabling sensitivity analysis. Data were analyzed using SPSS version 24. Secondary outcomes were also compared between cases and controls using WINPEPI [15].

## Results

Data were collected as follows: CLABSI (69 cases, 178 controls), CDI (92 cases, 276 controls) and SSI (76 cases, 215 controls). Seven CLABSI cases were excluded due to lack of control patients, leaving 62 CLABSI patients for analysis. Baseline characteristics were similar for cases and controls demonstrating successful matching (Table 1).

**Table 1:** Baseline patients’ characteristics (A-CLABSI, B-CDI, and C-SSI)

**A - Central-line associated blood-stream infection (CLABSI)**		
	**Cases**	**Controls**
	*N* = 62	*N* = 178
	N (%) / mean ± SD	N (%) / mean ± SD
**Demographics**		
Female gender	24 (39)	70 (39)
Age (years)	64.7 ± 16	64.2 ± 19
Age group (years)		
0–18 ^a^	18 (29)	54 (31)
> 18–50	10 (16)	27 (15)
> 50–70	14 (23)	41 (23)
> 70	20 (32)	55 (31)
Nursing-home residence	10 (16)	22 (12)
ICU type		
General (medical or surgical)	41 (66)	115 (65)
Cardiac or cardiothoracic surgery	3 (5)	9 (5)
Neonatal	18 (29)	54 (30)
Time from ICU admission to inclusion (days)	12 ± 17	11 ± 15
**Clinical characteristics at inclusion**		
Mechanical ventilation	40 (64)	114 (64)
Diabetes mellitus	17 (27)	52 (29)
Creatinine above 150 mmol/liter	17 (27)	51 (28)
Dialysis	5 (8)	10 (6)
Congestive heart failure	8 (13)	27 (15)
Cirrhosis	1 (2)	8 (4)
Albumin (g/L)	22± 5	26 ± 7
Chronic obstructive pulmonary disease (COPD)	5 (8)	15 (8)
Simplified Acute Physiology Score (SAPS) II		
≤ 52	12 (29%)	36 (30%)
> 52	40 (71%)	84 (70%)
**B -*Clostridium difficile*infection (CDI)**		
	**Cases**	**Controls**
	*N* = 92	*N* = 276
	N (%) / mean ± SD	N (%) / mean ± SD
**Demographics**		
Internal Medicine / Surgical ward	72:20 (78:22)	216:60 (78:22)
Female gender	49 (53)	147 (53)
Age (years)	76 ± 14	74 ± 14
Age group (years)		
> 18–50	6 (7)	18 (7)
> 50–70	15 (16)	45 (16)
> 70	71 (77)	213 (77)
Nursing-home residence	30 (33)	42 (15)
Time from admission to inclusion (days)	15 ± 17	14 ± 16
**Clinical characteristics at inclusion**		
Mechanical ventilation	3 (3)	9 (3)
Diabetes mellitus	34 (37)	132 (48)
Creatinine above 150 mmol/liter	33 (36)	87 (32)
Dialysis	6 (7)	20 (7)
Congestive heart failure	23 (25)	76 (28)
Cirrhosis	3 (3)	12 (4)
Albumin (g/L)	26 ± 6	30 ± 7
Chronic obstructive pulmonary disease (COPD)	6 (7)	46 (17)
Charlson comorbidity index		
≥ 6	64 (70)	192 (70)
**C - Surgical site infection (SSI)**		
	**Cases**	**Controls**
	*N* = 76	*N* = 215
	N (%) / mean ± SD	N (%) / mean ± SD
**Demographics**		
Deep: Superficial	39:37 (51:49)	–
Female gender	42 (55)	119 (55)
Age (years)	64 ± 18	64 ± 17
Age group (years)		
> 18–50	18 (24)	48 (22)
> 50–70	24 (32)	78 (36)
> 70	34 (45)	89 (41)
Nursing-home residence	0 (0)	6 (3)
Operation type		
Breast	4 (5)	12 (6)
Cholecystectomy	10 (13)	30 (14)
Colon	19 (25)	50 (23)
Inguinal hernia	5 (7)	15 (7)
Spine	10 (13)	29 (13)
Total hip replacement (THR)	11 (14)	30 (14)
Total knee replacement (TKR)	17 (22)	49 (23)
**Clinical characteristics at inclusion**		
Mechanical ventilation	0	0
Diabetes mellitus	13 (17)	26 (12)
Creatinine above 150 mmol/liter	4 (5)	1 (0.5)
Dialysis	1 (1)	0
Congestive heart failure	3 (4)	5 (2)
Cirrhosis	0	1 (0.5)
Albumin (g/L)	38 ± 7	40 ± 6
Chronic obstructive pulmonary disease (COPD)	2 (3)	4 (2)
American Society of Anesthesiologists (ASA) score		
1	13 (17)	33 (16)
2	43 (57)	137 (64)
3	19 (25)	39 (20)
4	1 (1)	2 (1)
5	0	0
Emergency	6 (8)	11 (5)

^a^ Among neonates: gestational age (weeks) was 30.8 + 5.7 among cases and 30.9 + 6.4 among controls; birth weight (grams) was 1750 ± 1465 among cases and 1660 ± 1244 among controls

### Hospital costs

#### Resource consumption

Use of blood tests, cultures, scans, duration of antibiotic treatment, length of stay and overall costs during 1st hospitalization were significantly higher for HAI patients than controls (Table 2). For CLABSI and CDI expenses per day were similar for cases and controls, as both costs and overall length of stay were higher. Costs per day were not calculated for SSI as reimbursement was determined according to DRG, not per diem.

**Table 2:** **Matched comparisons of resource consumption and costs, during 1st hospitalization, between patients with healthcare acquired infection and their controls**

A - Central-line associated blood-stream infection (CLABSI)				
	Cases	Controls	Difference	***p***-value
	*N* = 62	*N* = 178		
	Mean	Mean (min, max)^b^	Mean (min, max)^b^	Mean (min, max)^b^
Antibiotic days	34	13 (4, 23)	21 (11, 30)	< 0.001 (< 0.001, 0.095)
Imaging (number)	18	6 (2, 10)	12 (8, 16)	< 0.001 (< 0.001, 0.003)
Cultures (number)	16	5 (1, 10)	11 (6, 15)	< 0.001 (< 0.001, 0.003)
Blood tests (number)	115	42 (15, 77)	73 (38, 100)	< 0.001 (< 0.001, 0.015)
Hospitalization days since inclusion	34	14 (4, 25)	20 (9, 30)	< 0.001 (< 0.001, 0.2)
ICU days since inclusion	22	3.9 (1, 7.3)	18 (14.6, 20.1)	< 0.001 (< 0.001, 0.003)
Antibiotic cost (USD)	683	248 (52,572)	435 (111,631)	0.009 (< 0.001, 0.6)
Total cost ^a^ (USD)	6400	2376 (784,4512)	4024 (1888,5616)	< 0.001 (< 0.001, 0.05)
Total cost per day (USD)	601	719 (157,1614)	− 118 (− 1013,444)	0.63 (0.02, 0.02)
**B –*Clostridium difficile*infection**				
	**Cases**	**Controls**	**Difference**	***p*-value**
	*N* = 92	*N* = 276		
	Mean	Mean (min, max)^b^	Mean (min, max)^b^	Mean (min, max)^b^
Antibiotic days	18	6 (1, 13)	12 (5, 17)	< 0.001 (< 0.001, 0.1)
Imaging (number)	3.1	1.7 (0.3, 3.4)	1.4 (−0.3, 2.8)	0.11 (0.002, 0.71)
Cultures (number)	5.1	1.7 (0.24, 3.8)	3.4 (1.3, 4.86)	0.003 (< 0.001, 0.26)
Blood tests (number)	33	16 (5.5, 28)	17 (5, 27.5)	0.02 (< 0.001, 0.54)
Hospitalization days since inclusion	16.4	8.6 (4, 14.2)	7.8 (2.2, 12.4)	0.02 (< 0.001, 0.53)
Antibiotic cost (USD)	202	88 (6,215)	114 (−13,196)	0.009 (< 0.001, 0.85)
Total cost ^a^ (USD)	1357	733 (204,1426)	624 (−69,1153)	0.047 (< 0.001, 0.83)
Total cost per day (USD)	101	93 (32,174)	8 (−73,69)	0.5 (< 0.001, 0.001)
**C- Surgical site infection**				
	**Cases**	**Controls**	**Difference**	***p*-value**
	*N* = 76	*N* = 215		
	Mean	Mean (min, max)^b^	Mean (min, max)^b^	Mean (min, max)^b^
Antibiotic days	14.5	3.1 (1.9, 4.6)	11.4 (9.9, 12.6)	< 0.001 (< 0.001, < 0.001)
Imaging (number)	5.2	2.3 (1.2, 3.6)	2.9 (1.6, 4)	< 0.001 (< 0.001, 0.002)
Cultures (number)	3.6	0.58 (0.16, 1.2)	3.02 (2.4, 3.44)	< 0.001 (< 0.001, < 0.001)
Blood tests (number)	25	9.6 (6, 14)	15.4 (11, 19)	< 0.001 (< 0.001, < 0.001)
Hospitalization days since inclusion	8.3	5.5 (4.7, 6.4)	2.8 (1.9, 3.6)	0.002 (< 0.001, 0.032)
Antibiotic cost (USD)	148	18 (9,30)	130 (118,139)	< 0.001 (< 0.001, 0.001)
Total cost ^a,c^ (USD)	6761	5860 (5701,6071)	901 (690,1060)	< 0.001 (< 0.001, < 0.001)
Deep infection (*n* = 39)	8332	6852 (6697,7033)	1480 (1299,1635)	< 0.001 (< 0.001; < 0.001)
Superficial infection (*n* = 37)	5104	4815 (4652,5056)	289 (48,452)	0.04 (0.03¸ 0.56)

^a^Cost does not include fixed expense

^b^ min, mean of matched control patients with minimum resource consumption; max, mean of matched control patients with maximum resource consumption

^c^ Including the cost of the operation

*ICU* intensive-care unit, *USD* US Dollar

Sensitivity analyses comparing case patients to the control patients with the lowest resource utilization, demonstrated that resource utilization was significantly lower for all three HAIs. For control patients with the highest resource utilization, comparison was more variable and dependent both on type of infection and parameter compared (Table 2).

#### Opportunity costs

Length of ICU stay was 18 (±32) days longer for CLABSI patients vs controls (*p* < 0.001). As the first 4 days of ICU admission are reimbursed at a higher rate than subsequent ICU days, the longer ICU stay could represent lost income for the hospital (by preventing admission of other ICU patients with higher reimbursement). This represented an opportunity cost of $24,500 per CLABSI case (Table 4).

SSI patients were hospitalized for an additional 2.8 ± 7.6 days (mean ± SD), representing 50% of the mean length of stay per DRG case. During these additional days, the hospital could not admit another DRG patient representing an opportunity cost of $2581 per SSI case.

#### Readmissions

The hospital bears the cost of readmissions within 7 days of discharge of DRG patients. Of the 76 SSI patients (all DRG), 18 (24%) were readmitted within 7 days compared to 12/215 (6%) control patients (*p* < 0.001). The additional costs generated by readmissions averaged over all SSI patients, were $1469 per patient (Table 4). Readmissions for CLABSI and CDI patients incurred costs only for the insurer and are discussed below.

### Insurer costs

Insurer costs resulted from per diem reimbursement to the hospital for CLABSI and CDI patients/controls or DRG for patients/controls undergoing surgery. During first admissions, costs to the insurer were significantly higher for CLABSI and CDI patients, due to increased lengths of stay (Table 3). SSI patients also had increased length of stay; however the insurer was protected from increased costs by DRG payment.

**Table 3:** Insurer payment to hospital during primary hospitalization: matched comparisons between patients with healthcare acquired infection and their controls

	Cases	Controls	Difference	***p***-value
	USD			
	Mean	Mean (min, max)^c^	Mean (min, max)^c^	Mean (min, max)^c^
CLABSI^a^	24,673	10,065 (3281,24,673)	14,608 (0,21,392)	< 0.001 (< 0.001, 0.18)
CDI^a^	11,468	6038 (2795,9895)	5430 (1573,8673)	0.02 (< 0.001, 0.53)
SSI ^b^	9819	9793 (9749,9792)	26 (−40, 70)	0.77 (0.54, 0.54)

^a^ Hospitalization-days × cost per day

^b^ Diagnosis-related group (DRG)

^c^ min, mean of matched control patients with minimum resource consumption; max, mean of matched control patients with maximum resource consumption

*USD* US Dollar, *CLABSI* central-line associated bloodstream infection, *CDI Clostridium difficile* infection, *SSI* surgical site infection

Readmission within 90 days for CDI patients (42/92, 46% case patients vs 85/276, 31% control patients), and readmission of SSI patients between days 8 and 90 after discharge (47/76, 62% case patients vs 32/215, 15% control patients,) generated additional costs for the insurer, which were higher for cases than controls (Table 4).

**Table 4:** Summary of extra costs per hospital acquired infection/per patient^a^ for the hospital and insurer

	1st hospitalization Direct extra cost	Extra cost and opportunity cost due to extra hospital days (number of extra days)	Extra cost due to Readmission	Total extra cost per case
**Hospital**	USD			
CLABSI	− 2360^a^	24,585 (18.1)^b^	0	22,225
CDI	62^a^	0 (7.8)	0	62
SSI	900	2581 (2.8)	1469 ^c^	4951
**Insurer**				
CLABSI	–	14,608 (18.1)	− 1201	13,408
CDI	–	5430 (7.8)	5290	10,720
SSI	–	0 (2.8)	2395^c^	2395

^a^ We present the average cost difference per day multiplied by the average extra in-hospital days. The difference was NOT statistically significant and we only present the crude results

^b^ Extra potential loss due to higher charge for the first 4 ICU days (extra 1360 USD per day)

^c^ Hospital cost due to readmission within 1 week of discharge; Insurer cost due to readmission after 1 week of discharge

*USD* US Dollar, *CLABSI* central-line associated bloodstream infection, *CDI* clostridium difficile infection, *SSI* surgical site infection

In contrast, attributable costs associated with readmission for CLABSI patients (cases vs controls [discharged alive from first hospital stay] 5/27 (18%) vs 31/103 (30%)) were lower for cases than controls, as the length of readmission for cases was shorter than for controls (Tables 3 and 4).

### Secondary outcomes

Mortality within 90 days was similar for CLABSI cases and controls (36/62, 58% vs 87/178, 49%, *p* = 0.2). For CDI cases, 90 day mortality was significantly higher than controls (43/92, 47% vs 61/276, 22%, *P* < 0.001). Mortality for SSI was low for both cases and controls. Additional non cost-related outcomes are shown in Table 5.

**Table 5:** Secondary outcomes: mortality, discharge and 90 day readmission

	CLABSI N (%)		CDI N (%)		SSI N (%)	
	Case *N* = 62	Control *N* = 178	Case *N* = 92	Control *N* = 276	Case *N* = 76	Control *N* = 215
Discharge to LTCF	12 (19)	27 (15)	33 (36)	75 (27)	8 (11)	18 (8)
Hospital Mortality for first admission	35 (56)	75 (42)^$^	24 (26)	30 (11)^$^	1 (1)	1 (0.5)^$$$^
Mortality within 90 days	36 (58)	87 (49)^$$^	43 (47)	61 (22) ^$^	2 (2)	1 (0.5)^$$$^
90 days readmission^a^	5/27 (18%)	31/103 (30%)	42/68 (62%)	85/246 (35%)	47/75 (62%)^b^	32/214 (15%)^$, c^
Mean length of stay per patient during readmission^a^	1.5	4.3	16	4.4	7	0.5

^a^ Readmission data per number of patients discharged alive from first hospital stay

^b^ Seven patients (9%) required surgery during re-admission

^c^ Eight patients (4%) required surgery during re-admission

*CLABSI* central-line associated bloodstream infection, *CDI* clostridium difficile infection, *SSI* surgical site infection, *LTCF* long term care facility

Comparing cases to control $ *p* < 0.001, $$ *p* = 0.002, $$$ *p* = 1.0

## Discussion

Compared to well matched-controls, CLABSI, CDI and SSI were associated with increased resource use and total costs in a large academic medical center in Israel. However, these infections were also associated with longer admissions, resulting in similar costs-per-day for CLABSI and CDI cases and controls. These findings were consistent in almost all sensitivity analyses. As the hospitals are remunerated on a per diem basis for patients with these infections, there is no direct financial benefit to the hospital in preventing these infections. In contrast, as the hospital is remunerated on a per-patient and not per-day basis for elective surgical patients, there is a clear financial incentive to reduce SSIs. Adding analyses of opportunity and readmission costs slightly changes these findings, indicating a potential financial benefit in preventing CLABSI and SSI, but not CDI. For the insurer, preventing infection is always financially beneficial. Our data are analysed from the perspective of an independent hospital reimbursed from health care maintenance organizations, but the source data regarding costs etc. provide a generalizable base which could be adapted to other reimbursement strategies.

The analysis does include assumptions, such as that the hospital is always fully occupied, that demand for ICU beds exceeds supply, and that there are waiting lists for elective surgery. These assumptions reflect the reality in our country, but might not be accurate in other circumstances. These data are also important as they show that it is not only the direct cost of treatment of acquired infections, but also the specifics of remuneration that determine the financial benefits from preventing infections. It could be suggested that hospitals manipulate lengths of stay for per diem cases in order to optimize income, while shortening DRG lengths of stay. Israel has one of the lowest hospital beds/population of the OECD and the wards are almost always overflowing [16]. For this reason we believe it is unlikely that hospital lengths of stay were extended for financial reasons. The analysis presented will clearly be influenced by occupancy pressure, and waiting lists for surgery. Further, resource utilization is often considered to be highest at the beginning of an admission. However, it also increases around other significant events (such as HAIs). Our study attempted to control for costs prior to the HAI by matching length of stay prior to the HAI.

Reported attributable costs associated with nosocomial infections range from $5734 to $22,939 for CLABSI, $5042 to $7179 for CDI and $10,443 to $25,546 for SSI – figures which are significantly higher than those in our study [2, 4, 17–22]. There are several reasons for these differences. First, in most US studies, attributable costs include both treatment and fixed costs. We included only treatment costs, as fixed costs (such as personnel and structural maintenance) do not change according to the incidence of infection. (ii) Most studies match controls by database diagnosis and compare costs for groups [23]. We matched three specific controls to each case patient. (iii) Other explanations for the differences in costs relate to which costs are included. For example in the US, consultations with other services represent an additional source of costs while in Israel consultations do not generate costs. (iv) Further, not all studies defined the focus of costs – the hospital, insurer or the health care regulator. Our study clearly defined costs by payer. This is important as the focus of costs will define where financial incentives can be used to justify investment in infection prevention. (v) Finally, consideration of readmission is not uniform. Readmission costs are particularly relevant to the insurer (apart from DRG based readmissions within 7 days). We included all readmissions within 90 days as this is the maximum follow up period for diagnosis of SSI after surgery [24].

Preventing infections is an imperative for all stake-holders in the health care system. Infections cost money and probably worsen outcome. US insurers have stopped paying for “preventable” ventilator associated pneumonia. Whether this has led to a greater effort to reduce VAP rates, or merely led to reclassification of VAP with other diagnoses to ensure remuneration, is unclear [10]. Additionally Calderwood et-al and Lee et-al showed that the policy of stopping reimbursement for HAIs had minimal impact on hospital reimbursement [25, 26]. The regulator represents an additional stake-holder whose interest should extend beyond costs alone - their mission should be to improve health care in general. In Israel, hospitals are rewarded by the Ministry of Health for advancing infection prevention programs [27]. While this certainly encourages hospitals to promote infection prevention activities, the effect of these rewards on infection rates is unknown. Further, the Ministry of Health publishes periodic between-hospital comparisons of quality measures including different HAIs. Although not a direct financial incentive, this too might encourage hospitals to improve.

### Limitations

The costs of the resources taken into account in our study are specific to Israel, and might be priced differently in other countries. To allow comparability in other countries we have included the resources used themselves (antibiotic days, diagnostic tests etc.) in each table. Further, the number of diagnostic tests and treatments per day were similar for case and control patients meaning that overall costs per day would be similar, regardless of specific pricing. Additionally, in different reimbursement systems the burden of extra costs might be divided differently between the hospital and the insurer. Our study includes two per diem reimbursed infections (CLABSI and CDI) and one DRG reimbursed infection (SSI). The aim of the study was to demonstrate the effect of reimbursement strategy on financial considerations for different HAIs. If the HAIs are reimbursed differently in different jurisdictions, then clearly the analysis would have to be adjusted. We present the local situation as a representative example.

## Conclusion

We have shown that nosocomial infections are associated with both increased resource utilization and increased length of stay. The distribution of the additional financial burden between hospital and insurer, however, depends on remuneration policy and other organizational considerations. In the Israeli health system, there are no financial incentives for hospitals to prevent CLABSI and CDI events. Whether financial incentives can be used to encourage hospitals to improve infection prevention practices can only be determined based on local health service organization.

## Data Availability

The datasets used and/or analysed during the current study are available from the corresponding author on reasonable request.

## Abbreviations

HAI: Hospital acquired infections
CLABSI: Central-line associated blood-stream infections
CDI: *Clostridium difficile* infection
SSI: Surgical site infections
DRG: Diagnosis related groups
USD: United States Dollars
NIS: New Israeli Shekels
ICU: Intensive care unit
